# Biomineralization and Properties of Guanine Crystals

**DOI:** 10.3390/molecules28166138

**Published:** 2023-08-19

**Authors:** Haoxin Hu, Rongrong Xue, Fenghua Chen

**Affiliations:** 1 College of Chemistry and Materials Science, Fujian Normal University, Fuzhou 350117, China; qsz20221478@student.fjnu.edu.cn; 2 School of Resources and Chemical Engineering, Sanming University, Sanming 365004, China

**Keywords:** guanine crystals, characteristic features, polymorphs, biomineralization principles

## Abstract

Guanine crystals with unique optical properties in organisms have been extensively studied and the biomineralization principles of guanine are being established. This review summarizes the fundamental physicochemical properties (solubility, tautomers, bands, and refractivity), polymorphs, morphology of biological and synthetic forms, and the reported biomineralization principles of guanine (selective recrystallization of amorphous precursor, preassembled scaffolds, additives, twinning, hypoxanthine doping, fluorescence, and assembly). The biomineralization principles of guanine will be helpful for the synthesis of guanine crystals with excellent properties and the design of functional organic materials for drugs, dyes, organic semiconductors, etc.

## 1. Introduction

Biomineralization principles mainly originated the biomineralization processes of inorganic biominerals, especially calcium ion biominerals, which have had a significant impact in the biomimetic design and synthesis of functional materials [1,2]. In recent years, organic biomineralization has gradually become a mainstream concern and the types of organic biominerals being studied are becoming more and more diverse, including guanine, uric acid, etc. [3]. Guanine, an important component of DNA and RNA, is a common metabolite during the degradation of nucleic acids. Guanine can be secreted as waste or deposited as biominerals if the organism does not produce a guanase. Guanine crystals extracted from sea fish were the earliest pearlescent pigments. Guanine biominerals have been found in chordates, arthropods, mollusks, and bacteriophyta, and may be found in other phyla in the future [4]. The reported existing forms of guanine in organisms can be the anhydrous guanine β form [5], guanine monohydrate [6], or an amorphous phase [7].

The biological activity of guanine biominerals are highly related to optical functions in biological coloring and vision systems, including wide or narrow band reflectors, band adjustable reflectors, mirrors, stimuli-responsive structure colors, etc. [4,8]. The excellent optical properties usually stem from the thin microplatelet morphology of the anhydrous guanine β form which has a high refractive index (>1.8) of (100) planes and hierarchical assemblies (orderly and chaotic one-dimensional photonic crystal) [9]. Controlling guanine crystal polymorphism, morphology, size, and assembly can result in a wide array of different guanine biomineral properties and makes them so fascinating. For example, although the basic units are all thin hexagonal guanine platelets exposing (100) planes, photonic crystals based on guanine platelets and cytoplasm show a silver color in the scales of koi carps and a blue color in neon tetra fish [10,11].

Currently, the research on guanine and other organic biominerals is mainly focused on the relationships between their structures and properties. The formation mechanism of guanine crystals and how the system controls the size, geometry, crystallography, and assembly of guanine crystals in vivo to achieve various optical functions, such as diffuse scattering, broadband reflectors, and tunable photonic crystals, have gradually become research hotspots in recent years [8]. Artificially synthesized guanine crystals have exhibited some of the basic properties of biological guanine crystals, including polymorphs, orientations, size, morphology, pearly luster, etc. [8]. It is an interesting challenge for us to obtain optical devices based on guanine crystals at the macroscopic scale.

In this review, the basic properties of guanine crystals are summarized, the biomineralization principles of guanine biominerals and synthesized guanine crystals are listed, and the challenges and prospects for synthetic guanine crystals are discussed. An understanding of the control over guanine crystal formation and properties in biominerals and in laboratory settings is necessary in order to design suitable methods for obtaining photonic crystals based on guanine crystals, as well as other functional organic materials for use as drugs, dyes, and organic semiconductors.

## 2. Physical and Chemical Properties of Guanine Crystals

### 2.1. Solubility

Overall, the solubility of guanine in water is very low [12], which has made guanine recrystallization difficult in bioinspired guanine synthesis. During guanine dissolution, guanine nanoparticles with a diameter of about 0.8 μm form, making the aqueous solubility of guanine puzzling and slightly higher than expected [13]. The aqueous solubility data of guanine at 25 °C reported in the literature or obtained in our laboratory are shown in Table 1. Only the aqueous solubility of commercial guanine (raw guanine) has been reported, and commercial guanine is usually a mixture of the anhydrous guanine β form (AG β) and anhydrous guanine α form (AG α). The solubility of polymorphs is always different, which plays an important role in drug polymorphism [14]. Thus, we tried to test the aqueous solubility of different guanine phases in our laboratory. Our unpublished experimental results indicated that the solubility order of the various guanine phases in water at the same temperature is amorphous guanine, guanine monohydrate (GM) >> AG β > AG α. GM is not stable at high temperatures or after immersion in water for a long period of time, transforming into a mixture of AG α and AG β [15]. Thus, the specific solubility of GM was not obtained in our experiments, but its solubility must be higher than that of AG β and AG α. AG β is stable in water even after being immersed for a very long period of time [16]. Hydrated guanine crystals have a greater solubility than anhydrous guanine, which is uncommon in molecular crystals [14] and might be caused by the guanine molecules existing as different tautomers in these guanine crystals.

The solubility of guanine in ethanol is lower than in water [16]. Guanine has a higher solubility in dimethyl sulfoxide (DMSO) and dimethylformamide; however, it is sparingly soluble [19]. Guanine can be dissolved in acidic or alkaline solutions in which guanine is ionized [20]. Because guanine hydrochloride and guanine sulfate are also insoluble in aqueous solution, the biomimetic crystallization system for guanine usually utilizes guanine powders dissolved in ammonia or NaOH solutions as the source of guanine.

### 2.2. Tautomers

**Figure 1:**
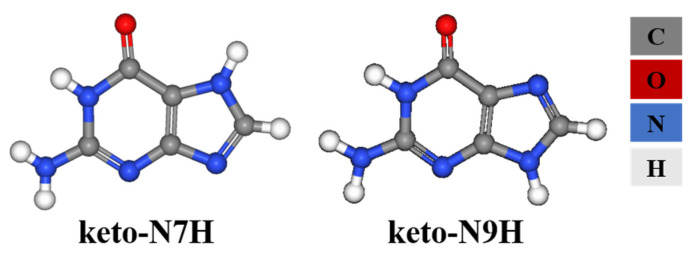
Molecular structure of keto-N9H and keto-N7H guanine tautomers that exist in guanine crystals.

Guanine has the most tautomers among the common nucleic acid bases. Guanine molecules have four acidic hydrogen atoms which can bind to seven sites (O and N atoms), producing 36 possible tautomers [21]. The reported guanine tautomers that exist in guanine crystals are keto-N9H and keto-N7H (Figure 1). The two keto amino forms are dominant in gases and solutions; keto-N7H is the most stable tautomer in gas, and keto-N9H is the most common type in aqueous solutions. The ratio of keto-N9H to keto-N7H increases with increasing solvent polarity [22].

Tautomeric polymorphs referred to the different crystal structures after tautomers crystallize [23]. Tautomers in the solid state have been referred to as desmotropy. Desmotropy (desmotropism) is when “at least two different tautomers of a molecule have been isolated in solid state” [24]. Tautomeric polymorphs include polymorphism (the same tautomer crystallizing in two or more crystal forms) and desmotropy. Only a very small number of tautomeric molecules have tautomeric polymorphs, and tautomeric molecules having desmotropy is rarer. Guanine is a very classical model of tautomeric polymorphs and desmotropy.

### 2.3. Band Gap and Refractivity

Guanine crystals with a direct gap of 3.60 eV are a promising wide-band gap semiconductor for optoelectronic devices [25]. Anhydrous guanine (including AG α and AG β) is composed of H-bonded layers, stacked along the ~3.6 Å translation axis; it is a monoclinic system and has strong anisotropic optical properties. Anhydrous guanine is a weak biaxial crystal with a refractive index of about 1.85, 1.81, and 1.46 [9]. The refractive index of anhydrous guanine in the normal direction of the (100) plane (~1.83) is one of the highest in biomaterials and much higher than that of water (~1.33).

## 3. Guanine Polymorphs and Morphology

**Figure 2:**
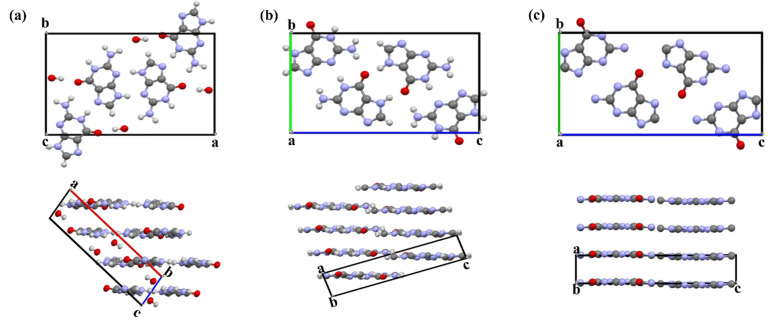
Crystal structures of (**a**) guanine monohydrate (GM), (**b**) anhydrous guanine α form (AG α), and (**c**) β form (AG β). These data are freely available from the Cambridge Crystallographic Data Centre (CCDC, www.ccdc.cam.ac.uk (accessed on 26 May 2023)).

Currently, three different anhydrous polymorphs and one monohydrate of guanine have been reported (Figure 2, Table 2 and Table 3), including AG α, AG β, dehydrated guanine monohydrate (dehydrated GM), and GM [8]. AG α (keto-N7H), AG β (keto-N7H), and dehydrated GM (keto-N9H) are desmotropy, and all the four polymorphs are tautomeric polymorphs. Moreover, a hydrated amorphous guanine (HAmG), obtained by a quick neutralization reaction and composed of keto-N7H, was confirmed by FT-IR, Raman, and ss-NMR characterization [16,26].

### 3.1. Guanine Monohydrate and Dehydrated Guanine Monohydrate

**Figure 3:**
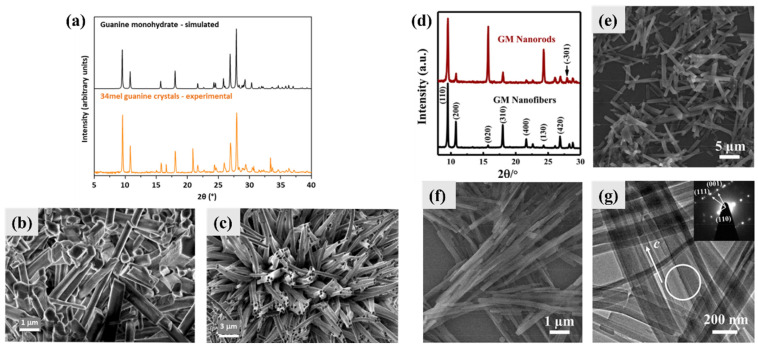
Typical biominerals and synthetic crystals of GM. (**a**) Powder X-ray diffraction (PXRD) pattern of GM produced by bacteria 34 mel and simulated PXRD pattern from single-crystal X-ray diffraction data. Scanning electron microscope (SEM) images of biological GM produced by 34 mel grown in a (**b**) solid and (**c**) liquid. Copyright 2023, BioMed Central [6]. (**d**) PXRD patterns of synthetic GM nanorods and nanofibers. (**e**) SEM image of GM nanorods obtained in phosphate-buffered solution (PBS) in the presence of 2.7 mmol·L^−1^ cetyltrimethyl ammonium bromide (CTAB). (**f**) SEM and (**g**) TEM images and the selected area electron diffraction (SAED) pattern of monocrystalline GM nanofibers obtained in an acidic solution. Copyright 2018, American Chemical Society [15].

GM (keto-N9H) was the first reported guanine crystal [27]. Preparation of pure-phase GM is not easy [15], and anhydrous guanine phases usually appear in the first several experiments to prepare GM according to our experience. The nucleation of GM becomes stable after multiple experiments, which is different from the phenomenon referred to as “disappeared polymorphism” [35]. The possible reason is that the GM particles that exist in a laboratory (in the vessel or air) would function as nucleation sites, which is similar to the explanations for disappeared polymorphism [35]. GM was reported to be present in the metabolites of some bacteria (Figure 3a) [6]. The typical morphology of GM in 34 mel bacteria is rods in solids and fibers in liquids (Figure 3b,c) [6]. The synthetic GM nanorods and nanofibers mainly showed (hk0) diffraction peaks (Figure 3d), indicating that the crystals’ growth orientation is (00l). The typical synthetic condition of GM nanorods is a neutralization process in neutral conditions in the presence of the surfactant cetyltrimethyl ammonium bromide (CTAB) (Figure 3e); these GM nanorods showed a (-301) diffraction peak (Figure 3d). The GM nanofibers were synthetized in an acidic solution (Figure 3f), and the orientation of the nanofibers was confirmed by selected area electron diffraction (SAED) (Figure 3g). Overall, the reported biominerals and synthetic crystals of GM are very similar in morphology. The current research on the synthesis of GM indicates that the formation of GM biominerals might be caused by acidic microenvironments and/or the presence of surface-active ingredients.

Dehydrated GM was prepared by heating GM at 150 °C [15]. The crystal structure of dehydrated GM is similar to that of GM with a greater shrinkage. Dehydrated GM is not stable at room temperature, and will transform into GM in a short period of time even under a low humidity condition. Dehydrated GM will transform into anhydrous guanine at a high temperature of 250 °C.

### 3.2. Anhydrous Guanine

The crystal structures of the AG α and AG β are very similar, and they have the same hydrogen-bonding network but different π-π stacking patterns. AG α and AG β are composed of the same hydrogen-bonded layers of guanine in the (100) crystallographic plane that stack along a*, whereas the layers are offset along the *c*-axis in AG α, and they are offset along the *b*-axis in AG β [5]. AG α is the most stable polymorph, but a single crystal of AG α was only prepared under harsh conditions [28]. AG β is a metastable phase and can remain stable for a relatively long period of time [16].

**Figure 4:**
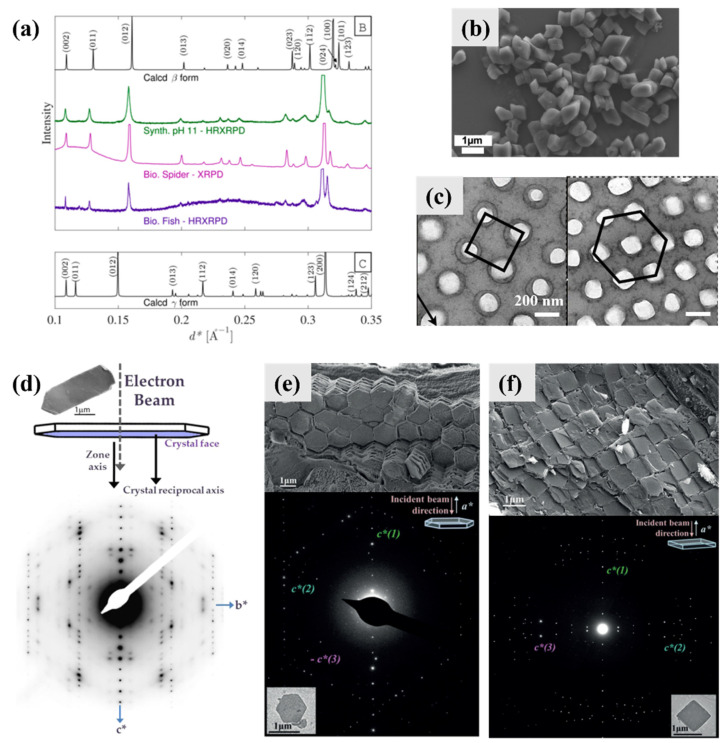
Typical biominerals of anhydrous guanine. (**a**) PXRD patterns of calculated, synthetic, or biogenic AG β and calculated AG γ. Copyright 2015, American Chemical Society [5]. (**b**) SEM image of guanine crystals extracted from the matt-white colored spider *Latrodectus pallidus*. Copyright 2010, Wiley-VCH [37]. (**c**) TEM images of guanine nanocrystals in superficial iridophores in the excited state in panther chameleons. Copyright 2015, Macmillan [38]. (**d**) TEM image of typical fish plate-shaped monocrystalline anhydrous guanine: a schematic representation of the guanine platelet in the TEM setup in which electron beam is perpendicular to the plate face and parallel to the proposed crystal zone axis, and the observed electron diffraction (ED) pattern. Copyright 2015, American Chemical Society [5]. Cryo-SEM images (top) and SAED patterns (bottom) of (**e**) the hexagonal guanine tiling covering the body of the *S. metallina* copepod and (**f**) a congruent layer of square guanine tiles taken from *P. maximus* scallop eye. The morphology and orientation of the measured crystal is displayed at the bottom corner of each ED pattern. A schematic view of the TEM setup is shown in the upper right corner of each ED pattern. Copyright 2017, Wiley-VCH [19].

AG β has been reported to be found in a variety of reptiles (including spiders, lizards, and chameleons) and fish (Figure 4a). The thermodynamic morphology of AG β (AG α) is prismatic crystals [4,29], which were also observed in guanine biominerals extracted from the matt-white-colored spider *Latrodectus pallidus* (Figure 4b). The three-dimensional lattice of guanine nanocrystals in the dermal superficial iridophores of panther chameleons has the potential to behave as photonic crystals; thus, the panther chameleon can change its color through modifying the guanine nanocrystal lattice (Figure 4c).

Platelets including monocrystalline platelets and twinned platelets are the most widespread morphology of guanine biominerals, which have been reported in fish, copepods, scallop, frogs, etc. [4]. Guanine platelets in fish (scales and eyes) are hexagonal plates, elongated along the *b*-axis and exposing the (100) plane (Figure 4d). The electron diffraction (ED) pattern of fish guanine platelets displays *mm* Laue symmetry and is consistent with a crystal zone axis parallel to the electron beam, which is perpendicular to the (100) face of the crystal. Thus, the proposed zone axis must be parallel to the crystal reciprocal axis that is normal to the crystal face. The ED pattern is in an orthorhombic arrangement, which is inconsistent with monoclinic AG β. AG γ (Figure 4a, Table 2), which has very similar crystal structures to AG β, was proposed to reconcile the contradiction [5]. The hydrogen-bonded layers in AG γ are offset in a “zigzag” mode, while in AG β the offset is of the “staircase” mode [5]. In our viewpoints, the proposed AG γ is one type of twinning of AG β. Overall, the ED of guanine platelets can be analyzed as an orthorhombic system.

Guanine platelets in copepod cuticles are almost perfect hexagonal plates, exposing the (100) planes (Figure 4e). Three *c*-axes were observed in copepod guanine crystals, while the electron beam was parallel to the *a*-axis of the crystals. The angles between two *c*-axes were 63 ± 1°, 61 ± 1°, and 56 ± 2°. Guanine platelets in scallop eyes are square (in fact, rhombic) plates that expose the (100) planes (Figure 4f). Similarly, three *c*-axes were observed in scallop guanine crystals, while the electron beam was parallel to the *a*-axis of the crystals. But the angles between two *c*-axis were 83 ± 0.5°, 83 ± 0.5°, and 14 ± 0.2°. Two different twinned guanine platelets have been observed in organisms so far. The morphology of both twinned guanine platelets is suitable to tile a non-defective mirror over a large area, which can reduce optical aberrations [36].

**Figure 5:**
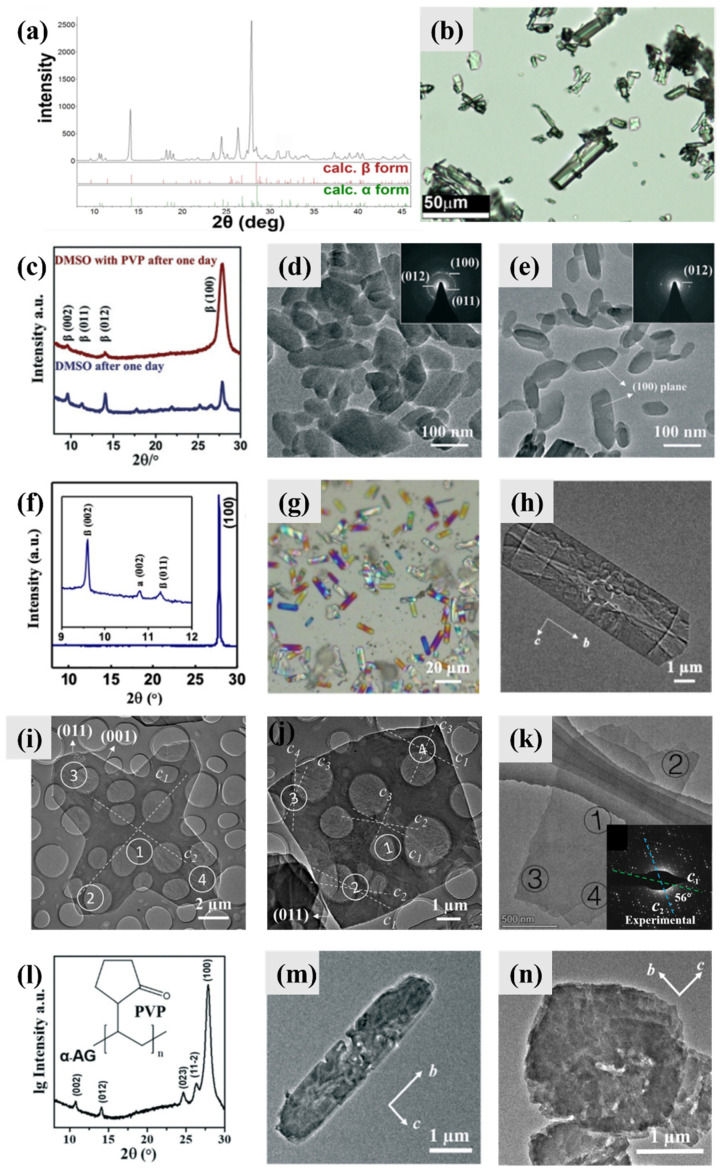
Typical synthetic anhydrous guanine crystals (pure phase instead of solid solution). (**a**) PXRD pattern of a mixture of AG α and AG β crystallized from guanine aqueous solutions (pH = 11), (**b**) light microscope image of prismatic crystals obtained from neutral and basic guanine aqueous solutions. Copyright 2016, American Chemical Society [20]. (**c**) PXRD patterns of AG β obtained via a hydrous amorphous guanine precursor in DMSO in the presence and absence of polyvinylpyrrolidone (PVP) after crystallization for one day. TEM images and SAED patterns (inset) of (**d**) irregular AG β nanocrystals without PVP and (**e**) AG β nanoplatelets in the presence of PVP. Copyright 2019, Royal Society of Chemistry [16]. (**f**) PXRD pattern of synthetic guanine microplatelets in formamide with poly (1-vinylpyrrolidone-co-vinyl acetate) (P(VP-co-VA)), indicating that the products are composed of almost all AG β crystals with a preferred (100) orientation. (**g**) Light microscope image of synthetic guanine microplatelets with beautiful structure colors. (**h**) TEM image of synthetic guanine microplatelets with annotated *b*- and *c*-axes. Copyright 2020, Wiley-VCH [31]. TEM images of (**i**) cross-shaped and (**j**) square-shaped synthetic twinned crystalline guanine microplatelets in formamide and water (volume ratio of 2:1) with P(VP-co-VA). Two *c*-axes with an angle of 84 ± 2° were found in the cross-shaped crystal, and four *c*-axes with angles of 19°, 85°, and 21° between the *c_1_*-axis and the other *c*-axes (*c_2_*, *c_3_*, *c_4_*) were found in the square-shaped crystal. Copyright 2019, Royal Society of Chemistry [32]. (**k**) TEM image of synthetic twinning guanine nanoplatelets in aqueous solution in the presence of P(VP-co-VA) via ammonia volatilization when stirred in an open state for 2 h and undisturbed in a sealed state for 4 h at 35 °C. Inset: SAED pattern of location 1 shows the two *c*-axes with an angel of 56°. Copyright 2023, Royal Society of Chemistry [39]. (**l**) PXRD pattern and (**m**) TEM image of synthetic AG α microplatelets in DMSO/water mixed solvent (volume ratio of 5:1) with PVP. (**n**) TEM image of synthetic crystals in the above conditions with hypoxanthine as an additive (molar ratios of guanine and hypoxanthine: 1.1). Copyright 2022, Royal Society of Chemistry [30].

Anhydrous guanine crystals synthesized under different experimental conditions show different morphologies (Figure 5). Thermodynamically stable prismatic anhydrous guanine crystals are also easy to obtain in laboratory (Figure 5a,b) [20]. In the absence of additives, the growth orientations of guanine crystals obtained through a neutralization reaction in aqueous solutions is along the *a*-axis.

Nanoprisms and nanoplatelets of AG β were obtained from recrystallization of HAmG in DMSO (Figure 5c–e). Both the PXRD and SAED patterns showed the different orientations of nanoprisms and nanoplatelets. The only difference in the recrystallization experiments was that polyvinylpyrrolidone (PVP) was added during the formation of the nanoplatelets.

A series of guanine microplatelets have been synthesized in recent studies. Until now, guanine crystals with sizes, morphologies, and crystallography that are mostly similar to those of biological guanine microplatelets found in fish were obtained through a one-step precipitation process using a guanine–sodium hydroxide solution in formamide in the presence of poly(1-vinylpyrrolidone-co-vinyl acetate) (P(VP-co-VA)) (Figure 5f–h); this process realizes the biomimetic synthesis of guanine crystals in size, polymorphs, and morphology at the same time [31]. The synthetic guanine microplatelets are mostly composed of AG β, confirmed by the PXRD pattern, and have a very strong preferred orientation that exposes the (100) plane, which was confirmed by the PXRD and SAED patterns. The light microscope image of synthetic guanine microplatelets showed beautiful structure colors, and the synthetic guanine microplatelets exhibited a prominent pearlescent luster when dispersed in an aqueous suspension, which is similar to the flashing structural colors of copepods [19].

Synthetic twinning AG β platelets were reported in two systems: a formamide/water (volume ratio of 2:1) system [32] and aqueous ammonia volatilization system [32,39]. The twinning with an angle of ~84° between the two *c*-axes in the cross-shaped and square-shaped crystals was the major form of twinning observed in the formamide/water system (Figure 5i,j), and a similar twinning angle was observed in the biogenic twinned guanine microplatelets in scallops. The twinning angle of ~20° between the *c*-axes was also observed in scallops, but a new twinning angle of ~45° between the *c*-axes appeared which was not observed in guanine biominerals. The twinning with an angle of ~84° was also commonly observed in the aqueous ammonia volatilization system; moreover, the twinning with an angle of ~60° between the *c*-axes was occasionally observed in the two systems (Figure 5k), which is similar to what is observed in copepods.

Interestingly, the microplatelets of AG α can also be easily obtained in a DMSO system [30] or formamide system [31], which indicates that guanine polymorphs are not the key factor of biological guanine morphology and AG α can also be used to construct optical materials. The microplatelets of AG α also showed a preference for exposing the (100) planes (Figure 5l) and the same *b*- and *c*-axes (Figure 5m). Except for the difference in polymorphs, the morphology and confused orthogonal ED pattern of AG α platelets were almost identical to those of biological and synthetic AG β platelets. Square AG α platelets (Figure 5n) were obtained in a DMSO/water mixed solvent (volume ratio of 5:1) in the presence of PVP and hypoxanthine, and hexagonal AG α platelets were obtained in formamide in the presence of P(VP-co-VA) and adenine. Both the square and hexagonal AG α platelets were monocrystalline instead of twinned. The twinning of AG α has not been observed so far, which might be due to the slow crystal growth rate under the conditions used to grow AG α.

## 4. Biomineralization Principles of Guanine Crystals

### 4.1. Intermediate Phase and Selective Recrystallization of Amorphous Guanine

**Figure 6:**
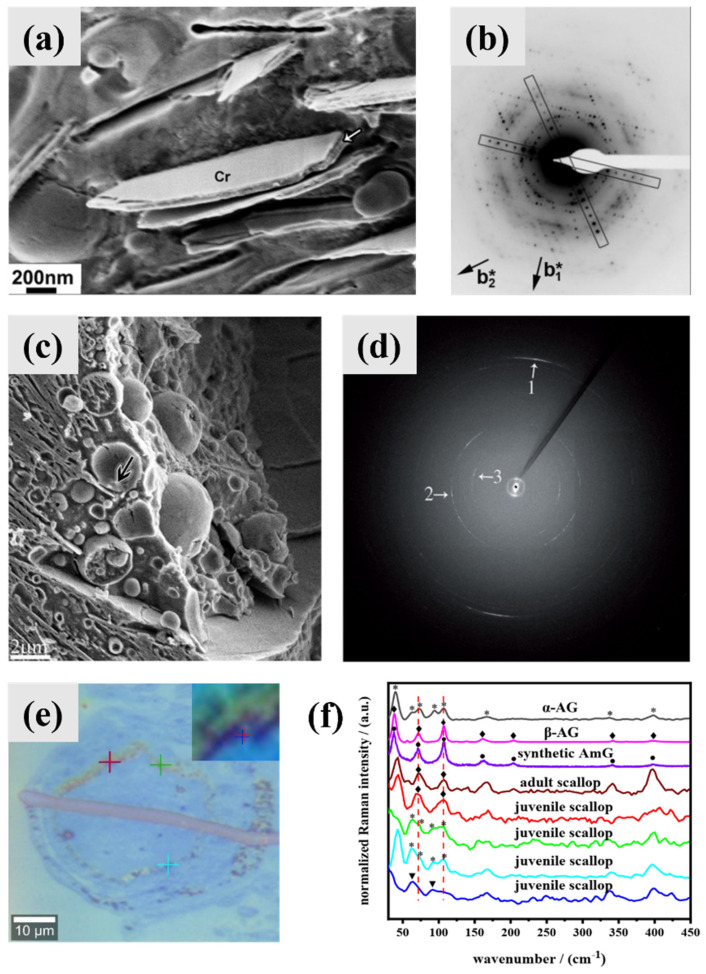
Amorphous guanine as intermediate phase in the formation process of guanine biominerals. (**a**) Cryo—SEM image of the typical crystal doublets (Cr) of the guanine platelets from silver-colored *Tetragnatha extensa* spider with a layer of a different material (suggested to be amorphous guanine) sandwiched between them (arrow). (**b**) ED pattern of the biogenic guanine crystals from *Tetragnatha montana* spider. The two ED patterns are essentially the same and correspond to the (100) zone axis of anhydrous guanine. Copyright 2010, Wiley-VCH [37]. (**c**) Cryo—SEM image after cryo—sectioning of the iridophores containing vesicles filled with a dense material (black arrows, suggested to be amorphous guanine). (**d**) XRD pattern obtained from a fixed Koi scale cross—section between hypodermis and epidermis: 1 and 2 correspond to the (102) and (012) reflections of AG, whereas 3 corresponds to the (110) reflection of GM. Copyright 2013, Wiley-VCH [7]. (**e**) Light microscopy image of the eyes of 2.5 mm *Patinopecten yessoensis* juvenile scallops and (**f**) low—frequency Raman spectra of AG α, AG β, amorphous guanine, and the concave mirror region of the eyes of adult scallops and juvenile scallops marked with different cross markers. Copyright 2022, bioRxiv [41].

Amorphous phases are important intermediate phases in the biomineralization of inorganic biological crystals, which has been a mature biomineralization principle [40]. Amorphous guanine intermediate phases were proposed to exist in the formation process of multiple guanine biominerals [7,37,41]; however, direct observation of amorphous guanine phases is lacked and only indirect evidence has been reported thus far. In the typical crystal doublets of guanine platelets from silver-colored spiders (Figure 6a,b), a layer of a different material (arrow) separating the two crystals within a doublet was proposed to be amorphous guanine not cytoplasm, which was supported by the fact that the material showed less etching than cytoplasm [37]. The iridophores of fish scales contain vesicles filled with a dense material (Figure 6c), which is much more resistant to water than the surrounding cytoplasm [7]. The X-ray diffraction patterns of the areas around the vesicles showed the signals of AG and GM at the same time (Figure 6d). As the biogenic crystals of fish are composed solely of AG, the GM formed from a pre-existing, non-diffracting source of guanine which was referred to as amorphous guanine [7].

Low-frequency Raman spectra (LFRS) are related to unit cell motions (lattice vibrational frequencies), and can be used to distinguish AG [42]. Raman peaks at 40 cm^−1^, 63 cm^−1^, 72 cm^−1^, 94 cm^−1^, and 107 cm^−1^ were assigned to AG α, and peaks at 39 cm^−1^, 72 cm^−1^, 107 cm^−1^, and 204 cm^−1^ were attributed to AG β [41]. The LFRS of the concave mirror region of the eyes of adult scallops only showed the signals of AG β, while that of juvenile scallops showed the signals of both AG α and AG β (Figure 6e,f) [41]. As the biogenic crystals from scallops are composed solely of AG β, AG α should form from a pre-existing amorphous guanine.

For guanine biominerals, the difficulty in observing amorphous guanine directly is due to the lack of technology which can provide information on the composition and amorphous phase at the same time in an accurate location; electron diffraction can nearly provide these two pieces of information of crystals at the same time.

**Figure 7:**
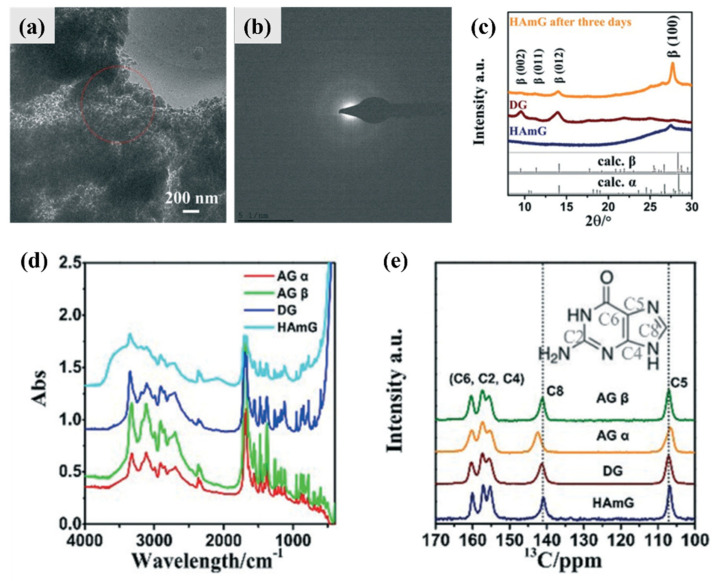
Selective crystallization behavior of hydrated amorphous guanine (HAmG) in neutral conditions and its explanation. (**a**) Cryo-TEM image and (**b**) SAED pattern taken from the area indicated with a red circle in (**a**) of HAmG obtained by a quick precipitation after mixing a basic aqueous solution with dissolved guanine and an H_2_SO_4_ solution. (**c**) PXRD patterns of freshly prepared HAmG, HAmG kept at RT for 3 days, and dried hydrous amorphous guanine (DG). (**d**) FT-IR spectra and (**e**) solid state nuclear magnetic resonance (ss-NMR) results of HAmG, DG, AG α, and AG β. Copyright 2019, Royal Society of Chemistry [16].

Only hydrous amorphous guanine consisting of keto-N7H was synthetized in neutral conditions by a quick precipitation after mixing a basic aqueous solution with guanine dissolved in an acidic solution [16]; anhydrous amorphous guanine or amorphous guanine containing keto-N9H have not yet been reported. Cryo-TEM imaging showed that the sample is composed of small isolated or aggregated spherical nanoparticles about 20 to 50 nm in diameter (Figure 7a), and the related SAED pattern exhibited no diffraction rings, confirming the amorphous feature (Figure 7b). The PXRD pattern of hydrous amorphous guanine showed a broad peak and a small peak at ~27.5° (Figure 7c), which is considered as interlayer π-π stacking of guanine molecules. Hydrous amorphous guanine is not stable and converts to the β form during the drying process or storage in water. The FT-IR spectra (Figure 7d) of the AG α, AG β, hydrous amorphous guanine, and dried hydrous amorphous guanine cannot be distinguished, indicating that the molecules of guanine in these phases are all keto-N7H. ^13^C ss-NMR spectra (Figure 7e) showed that the C8 shift of hydrous amorphous guanine was more similar to that of AG β (141.2 ppm) than AG α (142.4 ppm). Hydrous amorphous guanine has hydrogen bonding structures and π-π stacking layer structures (short-range orders) similar to AG β. The LFRS (Figure 6f) of hydrous amorphous guanine can be distinguished from that of AG α, but not AG β [41]. The short-range orders of amorphous guanine could be analyzed with ^13^C ss-NMR spectra and low-frequency Raman spectra until now.

**Figure 8:**
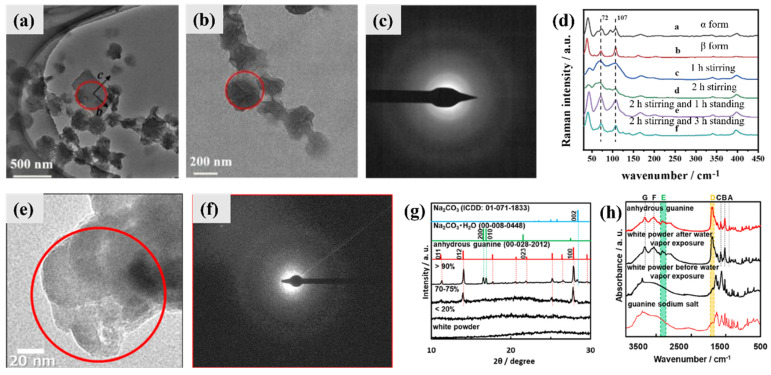
Selective crystallization behavior of amorphous phases containing guanine in alkaline conditions and its explanation. (**a**,**b**) TEM images and (**c**) SAED pattern of the products (mostly AG β, confirmed with PXRD pattern) obtained in aqueous solution in the presence of P(VP-co-VA) via ammonia volatilization and stirring for 1 h in an open state at 35 °C. (**d**) Low—frequency Raman spectra of AG α, AG β, and crystallization products obtained after 1 h of stirring in an open state, 2 h of stirring in an open state, 2 h of stirring in an open state and 1 h undisturbed in a sealed state, 2 h of stirring in an open state and 3 h undisturbed in a sealed state. All reactions were carried out at 35 °C. Copyright 2023, Royal Society of Chemistry [39]. (**e**) TEM image and (**f**) SAED pattern of the white powders obtained by freeze—drying an alkali aqueous solution with dissolved guanine. (**g**) PXRD patterns and (**h**) FT—IR spectra of the white powder before and after water vapor exposure. Standard PXRD patterns of AG β, sodium carbonate monohydrate, and sodium carbonate; standard FT-IR spectra of AG β and guanine sodium salt (heptahydrate disodium guanine) are also shown. Copyright 2020, American Chemical Society [43].

Some amorphous phases containing guanine were also observed in alkaline conditions. In the early stage (1 h) of the process of twinning guanine platelets in an aqueous ammonia volatilization system, mixed twinned guanine platelets and spherical particles were observed by TEM at the same time (Figure 8a,b) [39]. The spherical particles are the primary components of the samples, and the SAED pattern of the spherical particles showed amorphous features (Figure 8c) [39]. The LFRS of the products obtained in the early stage (1 h and 2h) showed broad peaks at 72 and 107 cm^−1^ (Figure 8d), which is much broader than that of AG β and was assigned to the spherical amorphous guanine particles. The two bands at 72 and 107 cm^−1^ in the spectra of the guanine samples obtained after 3 h and 5 h became sharper and stronger in comparison to those obtained at the early stage, which indicates that the amorphous spherical particles slowly transformed into crystalline AG β as the reaction time was extended [39].

Amorphous white powders were obtained by freeze-drying an alkali aqueous solution with dissolved guanine, and were confirmed by TEM with SAED and PXRD patterns (Figure 8e–g) [43]. The amorphous white powder was composed of small spherical particles 52 ± 10 nm in diameter. According to the PXRD patterns, the crystallized powder formed from the amorphous state at a relative humidity higher than 70% was AG β with sodium carbonate monohydrate and sodium carbonate (Figure 8g) [43]. The FT-IR spectrum of the white powder was similar to that of guanine sodium salt (heptahydrate disodium guanine), and the spectrum of the crystallized powder was similar to that of AG (Figure 8h) [43]. The results of energy-dispersive X-ray (EDX) analysis of the amorphous white powder showed that it was composed of carbon, nitrogen, oxygen, and sodium. The amorphous white powder was confirmed to be amorphous guanine sodium salt, which was produced through the rapid accumulation of the molecules during the freeze-drying process.

Both of the two different systems in alkaline conditions showed that amorphous intermediate phases containing guanine have a tendency to crystallize into AG β.

### 4.2. Preassembled Scaffolds and Interface Control

**Figure 9:**
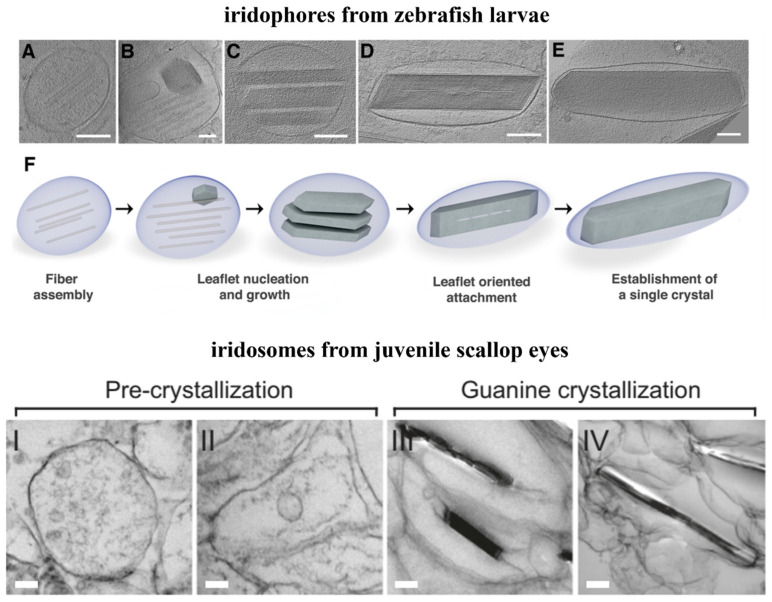
Preassembled scaffolds in the guanine iridophores. Top: iridophores isolated from zebrafish larvae, in which biogenic plate-like guanine crystals form via templated nucleation of (100) crystal leaflets on preassembled protein fibers. (**A**–**E**) CryoET reconstructions of iridophores isolated from zebrafish larvae at different maturation stages. Scale bars are 100 nm. (**A**) An early iridosome showing a scaffold of parallel protein fibers. (**B**) A more developed iridosome, with the initial nucleation of a (100) crystal leaflet on a protein scaffold taking place. (**C**) Several individual leaflets, imaged edge-on, with their (100) crystal plane parallel to each other. (**D**) The leaflets have almost completely coalesced into a single crystal. (**E**) A mature iridosome, where the leaflets have completely merged into a single crystal, with no obvious traces of the initial leaflets. (**F**) Schematic illustration of the proposed crystallization mechanism. Copyright 2022, American Chemical Society [45]. Bottom: iridosomes from juvenile scallop eyes. TEM images of iridosomes at different stages. Scale bars: 100 nm. Stage **I**: spherical iridosome containing intraluminal vesicles and disorganized fibrils. Stage **II**: iridosome elongates concomitantly with the formation of two fibrillar sheets extending across the organelle. Stage **III**: the sheets template the nucleation and growth of a guanine crystal and become bound to its (100) face. Stage **IV**: the growing guanine crystal stretches the organelle membrane tightly around itself. Copyright 2023, Macmillan [46].

Preassembled scaffolds based on a macromolecular matrix are widely found in biominerals. Typically, mollusks produce nacre by first generating several layers of an insoluble β-chitin matrix filled with silk fibroin gel, and then aragonite cores form on the surface of the matrix at the nucleation sites, followed by lateral growth in the confined space of adjacent organic layers, which finally leads to a Voronoi pattern [44]. A similar mesoscale “assembly-and-mineralization” approach, inspired by the natural process, was used to fabricate bulk synthetic nacre that highly resembles both the chemical composition and the hierarchical structure of natural nacre [44]. Recently, guanine platelets in zebrafish and scallop were also found to grow on preassembled scaffolds (Figure 9) [45,46]. Iridosomes isolated from the early cells of zebrafish larva contained up to 10, usually parallel, fibers that were approximately 20 nm in diameter and 200–400 nm in length (Figure 9A). The developed iridosomes revealed small crystals with a typical (100) plate-like morphology in close contact with the preassembled fiber scaffold (Figure 9B) [45]. Staining with Thioflavin T indicated that these fiber scaffolds are proteins, which aggregated as amyloid fibers [45]. Fast Fourier transform analysis on images of fiber-containing iridosomes revealed that the fibers had a periodicity of ∼1.9 nm, corresponding to the inter ribbon spacing of the β-sheet fibril [45]. Later, the elongating crystals reached the membrane and formed plate-like crystals via templated nucleation of thin leaflets on preassembled amyloid fiber scaffolds (Figure 9C). Following the alignment and gradual oriented attachment of crystal leaflets (Figure 9D), a single plate-like guanine crystal finally formed with the disappearance of the fibers (Figure 9E). The sequence of events is illustrated schematically in Figure 9F. The four stages of guanine morphogenesis formation show a resemblance to melanosome formation (Figure 9I–IV) [46]. In the early iridosome, intraluminal vesicles and disorganized fibrils are present (stage I). Prior to nucleation, two highly oriented intraluminal sheets assemble concomitantly and transform into an ellipsoid (stage II). The 2D sheets then template the nucleation and growth of guanine (stage III). Finally, following guanine deposition, the template sheets remain an integral part of the organelle and are fused to the guanine surface in iridosomes (stage IV). The existence of preassembled intraluminal slices inspired researchers to use the above mechanism for the synthesis of lamellar guanine crystals.

**Figure 10:**
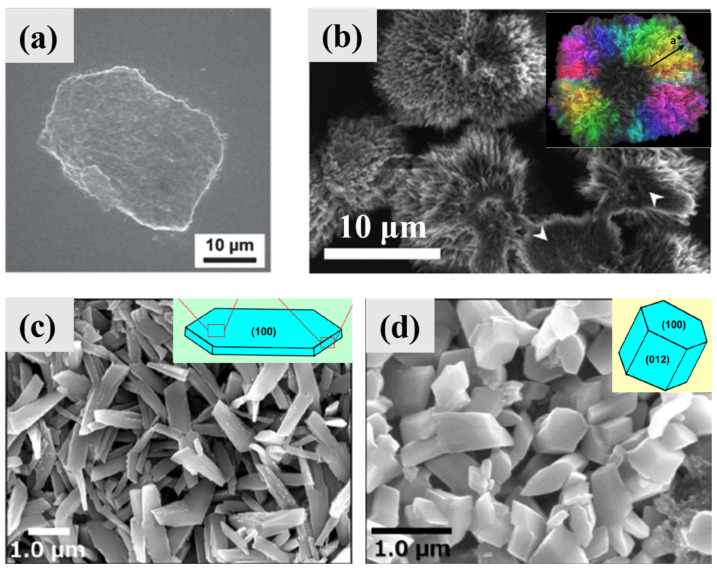
Interface effects on the formation of AG β. (**a**) SEM image of the platy guanine crystal formed on the chitosan substrate in the presence of poly-Glu through an aqueous ammonia volatilization method. Copyright 2012, Royal Society of Chemistry [33]. (**b**) SEM image of vase-like crystal aggregates formed by ammonia-induced crystallization of guanine-HCl solution (0.3 mg·mL^−1^) at the air–water interfaces. Inset hue saturation value (HSV) maps of azimuthal angle showing that the crystal long axes coincide with a*. Copyright 2020, American Chemical Society [34]. SEM images of the recrystallization products of amorphous guanine sodium salt (**c**) through two-dimensional growth with the enlargement of the hydrophobic (100) plane in thin water layers at a relative humidity of around 70% and (**d**) three-dimensional growth due to the π-π stacking of guanine molecules along the a-axis in a bulk liquid at a relative humidity greater than 90%. Copyright 2020, American Chemical Society [43].

The interface effects on the morphology of guanine crystals (AG β) in the laboratory is another major method of control. Platy guanine crystals can be obtained on chitosan substrates in the presence of poly-Glu through an aqueous ammonia volatilization method (Figure 10a) [33]. Vase-like guanine crystals were formed by ammonia-induced crystallization of a guanine-HCl solution at the air–water interfaces, and related hue saturation value (HSV) maps of the azimuthal angle showed that the crystals’ long axes coincide with a* (Figure 10b) [34]. The vase-like AG β crystals are similar to the bow-tie-shaped aggregates on glass substrates after aqueous ammonia volatilization [33]. Amorphous guanine sodium salts can recrystallize into biomimetic platy crystals in thin water layers at a relative humidity of around 70% through a two-dimensional growth mechanism (Figure 10c), or columnar crystals in a bulk liquid at a relative humidity greater than 90% through a three-dimensional growth mechanism (Figure 10d) [43].

### 4.3. Macromolecules and Small Organic Molecules as Additives

It is unknown how organisms control the polymorphs and morphology of guanine crystals at the same time. In the laboratory, polymers such as PVP, P(VP-co-VA), and poly(1-vinylpyrrolidone-co-2-dimethylaminoethyl methacrylate) (PVP-co-DM), and small organic molecules such as uric acid (UA), hypoxanthine (I), xanthine (X), adenine (A), and guanosine (GR) were used as additives to control the polymorphs and morphology of guanine crystals in different systems.

**Figure 11:**
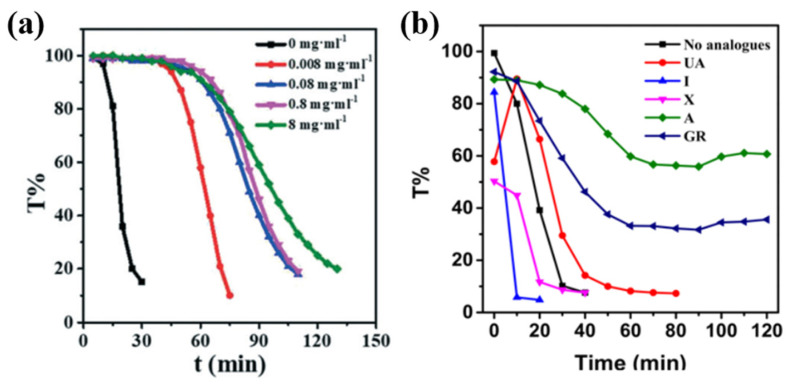
Investigation of the precipitation rate with different (**a**) PVP concentrations in the neutralization precipitation system in DMSO/water, Copyright 2022, Royal Society of Chemistry [30], and (**b**) excess guanine analogues including uric acid (UA), hypoxanthine (I), xanthine (X), adenine (A), and guanosine (GR) in the neutralization precipitation system in formamide with P(VP-co-VA), Copyright 2020, Wiley-VCH [31]. In situ turbidity measurement: the transmission intensity of the reaction solution at a wavelength of 500 nm was recorded as a function of reaction time.

The reaction kinetics have a profound effect in determining the polymorphs of anhydrous guanine. The precipitation rates were determined by tracking the transmittance intensity (T%) of the neutralization precipitation solutions in DMSO/water, with different PVP concentrations at 500 nm (Figure 11a) [30]. Without PVP, the products are a mixture of AG α and β, and with PVP (0.8 mg·ml^−1^), the products are the pure-phase of AG α [30]. The transmission results indicated that the precipitation induction time and precipitation rate of guanine decreased with the increase in PVP concentrations. PVP can inhibit the precipitation of anhydrous guanine and thus induce the formation of AG α in a thermodynamic manner. The kinetic meta-stable AG β is formed with a fast precipitation rate in the neutralization precipitation system in formamide with P(VP-co-VA) [31]. The products can maintain the β form with additives such as UA, I, and X, while it can change to the α form with additives such as A and GR [31]. In situ turbidity experiments revealed that the reaction rate in the presence of UA, I, and X is much faster compared with the reactions with A and GR (Figure 11b) [31]. The presence of UA, I, and X, similar to the case without analogues, leads to the formation of the metastable β form through a kinetically controlled crystallization process, and the presence of A and GR leads to the formation of a more thermodynamically stable α form through a thermodynamic crystallization process. The presence of I and X results in a faster reaction time than the presence of UA or without analogues, which leads the formation of the twining β form.

**Figure 12:**
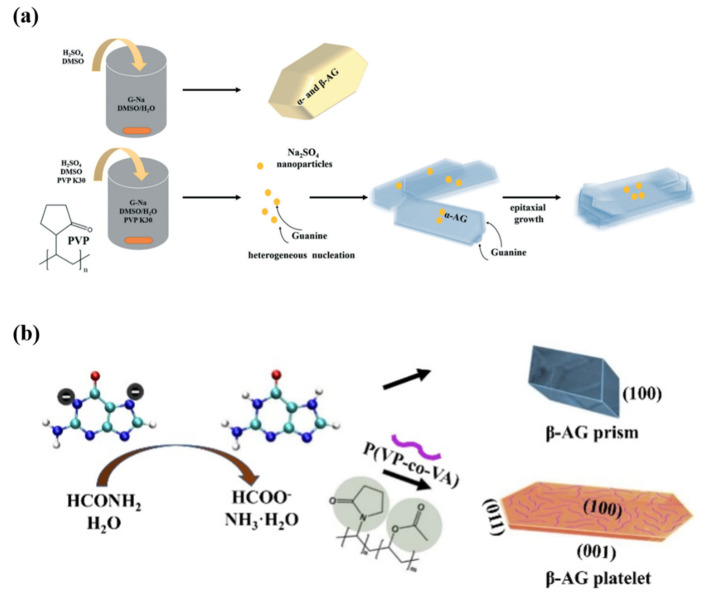
Schematic representation of (**a**) the possible formation mechanism of the AG α microplatelets in the neutralization precipitation system in DMSO/water, Copyright 2022, Royal Society of Chemistry [30], and (**b**) the possible formation mechanism of the AG β microplatelets in the neutralization precipitation system in formamide with P(VP-co-VA), Copyright 2020, Wiley-VCH [31].

At the current stage, the reason for the formation of the platelet morphology of the synthetic guanine crystals is believed to be due to the polymer PVP or P(VP-co-VA) having a tendency to adsorb onto the more hydrophobic (100) plane compared with other planes and it does not contribute to the hydrogen-bonding between guanine molecules within the (100) planes, thus resulting in the microplatelet morphology with the exposed (100) planes (Figure 12). The working concentration range of P(VP-co-VA) and PVP is wide (>three orders of magnitudes) to obtain the platelet morphology. Because the α and β forms are very similar to each other and both form π-π stacking interactions between the neighboring layers of the guanine molecules, it is reasonable that these polymer additives can absorb and stabilize the (100) planes of the α form (Figure 12a) and β form (Figure 12b).

### 4.4. Twinning

**Figure 13:**
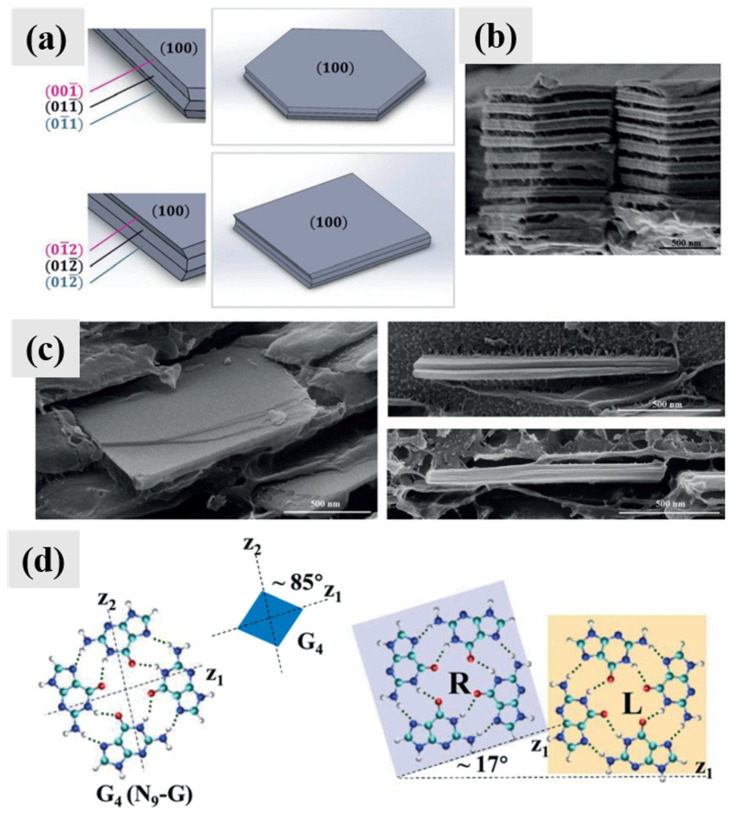
Twinning mechanisms proposed for biological and synthetic twinned guanine platelets. (**a**) Scaled geometrical models of the twinned hexagonal crystal morphologies in copepods and square-shaped crystal morphologies in scallops, supported by the edge-on cryo-SEM images of (**b**) the hexagonal and (**c**) the square-shaped crystals. Copyright 2017, Wiley-VCH [19]. (**d**) G4 (keto-N9H guanine) and its *R*- and *L*-type assemblies. The angle of ~85° is between the two vectors of G4, and the angle of 17° is between the directions of its *R*- and *L*-type assemblies. Copyright 2019, Royal Society of Chemistry [32].

A molecular interlayer twinning mechanism was proposed to explain the regular hexagonal or square-shaped crystal of monoclinic guanine (Figure 13a) [19]. For hexagonal guanine platelets in copepods, a {100} plate delimited by two {001} and four {011} lateral faces would therefore have a slightly distorted hexagonal morphology [19]. To generate a regular hexagonal crystal with six equivalent side faces, each side face was proposed to be composed of layers of {001} and {011} faces via a molecular interlayer twinning mechanism with two twinning operations (Figure 13a) [19]. For square-shaped guanine platelets in scallops, although the crystal with a {100} plate-like rhombus shape delineated only by four {012} faces is almost square, such a hypothetical morphology would not be formed normally. In fact, such a monocrystalline square-shaped platelet morphology of AG α was reported in DMSO and water (volume ratio of 5:1) with PVP and hypoxanthine (Figure 5n) [30]. To generate a twinned square-shaped crystal, two twinning operations were applied as a simulation (Figure 13a) [19]. The molecular interlayer twinning mechanism is supported by edge-on cryo-SEM images of the twinned crystal morphologies in copepods (Figure 13b) and scallops (Figure 13c), which show that each platelet is composed of different domains with evident interface exposure.

It has been reported that the morphology of synthetic twinned guanine platelets is largely similar to that of scallop guanine biominerals [32]. A mechanism based on guanine molecular assembly was proposed to explain the different twinning angles obtained in the synthetic twinned guanine microplatelets. The formation of synthetic twinned guanine microplatelets with angles of 84° and 20° between the *c* diffraction vectors might be related to the attachment of a G-quartet (G4, keto-N9H) molecular structure on the nuclei or freshly formed nanocrystals at the early stage of crystallization with certain directions [32]. The angle between the two axes of the G-quartet is about 85°. The G-quartet on the substrates has two kinds of chiral molecular structures, *R*-type and *L*-type, which are mirror structures of each other and the angles between the directions of the *R*- and *L*-type assemblies is 17°. Although the G-quartet is composed of keto-N9H, it is possible for the G-quartet to adsorb onto anhydrous guanine, similar to the assemblies of the G quartet–Na network (keto-N9H) structure with patches of the keto-N7H purine ring [47]. The formation of twinning angles of 60° might be caused by guanine assemblies similar to the hydrogen-bonding networks in GM, which has a ring composed of six guanine molecules (keto-N9H).

### 4.5. Solid Solutions/Hypoxanthine Doping

The molecular structure of hypoxanthine is similar to that of guanine, and only lacks an NH_2_ group. Guanine biominerals containing hypoxanthine are widely found in guanine-containing tissues in fish, frogs, and nudibranchs [48].

**Figure 14:**
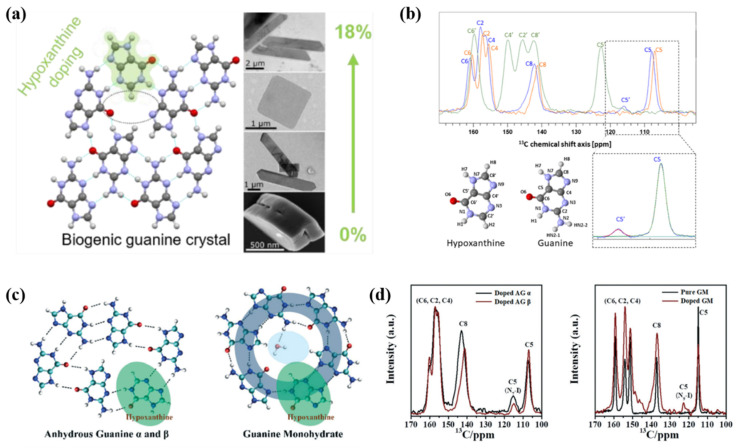
Solid solutions of guanine and hypoxanthine in biogenic guanine (AG β) and synthetic guanine (all the reported guanine phases). (**a**) Hydrogen-bonded layer of AG β with one guanine molecule substituted with a hypoxanthine molecule. SEM images from bottom to top correspond to prismatic guanine crystals from the white spider *L. pallidus* (the ratio of hypoxanthine in guanine biominerals, 0–2 mol%), elongated hexagonal crystal plates from the skin of the fish *D. labrax* (0–6 mol%), square-shaped plates from the eye of the scallop *P. maximus* (8 mol%), and elongated hexagonal crystal plates from the skin of the fish *S. salar* (13–18 mol%). (**b**) ^13^C CPMAS (cross-polarization magic angle spinning) spectra of the swim bladder crystals (blue), synthetic AG β (orange), and hypoxanthine crystals (green). The annotated guanine and hypoxanthine structures correspond to the spectral labeling. Copyright 2022, American Chemical Society [48]. (**c**) The hydrogen-bonding networks of AG α and AG β (left) and GM (right) doped with hypoxanthine. (**d**) ^13^C ss-NMR (solid state NMR) of various doped guanine phases including AG α, AG β, and GM. Copyright 2022, Royal Society of Chemistry [26].

Biogenic guanine crystals are not pure crystals but molecular alloys (solid solutions) of guanine, hypoxanthine, and sometimes xanthine. Due to the smaller molecular volume of hypoxanthine, hypoxanthine molecules can easily substitute guanine and dope into the H-bonding layer of AG β (Figure 14a). The molar ratio of hypoxanthine in biological guanine crystals are in the range of 0–18 mol%; for example, the ratio is 0–2 mol% in the prismatic guanine crystals from the white spider *L. pallidus*, 0–6 mol% in the elongated hexagonal crystal plates from the skin of the fish *D. labrax*, 8 mol% in the square-shaped plates from the eye of the scallop *P. maximus*, and 13–18 mol% in the elongated hexagonal crystal plates from the skin of the fish *S. salar* (Figure 14a) [48]. The ^13^C NMR spectrum of the swim bladder crystals mainly exhibits resonances (C5 of guanine at 107 ppm) attributable to AG β (Figure 14b). An additional peak at 116 ppm is attributed to the C5 of hypoxanthine, and is different from the C5 of anhydrous guanine and C5 of hypoxanthine at 123 ppm (Figure 14b), which confirmed the formation of solid solutions. Until now, no evidence has shown a correlation between the biogenic crystal morphology and dopant content [48]. In fact, hypoxanthine can be doped into all the reported guanine phases by solution methods [26], which shows that hypoxanthine is not the key factor in controlling the polymorphs of biogenic guanine.

Keto-N7H hypoxanthine can be doped into the hydrogen-bonding networks of HAmG, AG α and AG β form, and keto-N9H hypoxanthine can be doped into the hydrogen-bonding networks of GM and dehydrated GM (Figure 14c) [26]. The doping concentrations of hypoxanthine in these phases can all be over 10 wt.%. The existence forms of hypoxanthine in the different solid solutions were confirmed by ^13^C ss-NMR (Figure 14d) and mid-frequency Raman spectra. The peaks at 115 ppm in the ^13^C ss-NMR spectrum of the doped AG α and AG β were identified as the C5 sign of keto-N7H hypoxanthine. The peak at 123 ppm in the ^13^C ss-NMR spectrum of the doped GM is considered to be the C5 sign of keto-N9H hypoxanthine, which is similar to that in hypoxanthine crystals composed of keto-N9H hypoxanthine [26]. The hypoxanthine Raman bands at 722 cm^−1^ in the doped HAmG, 724 cm^−1^ in the doped AG α, and 725 cm^−1^ in the doped AG β were different from hypoxanthine Raman bands at 718 cm^−1^ in the doped GM, which also prove that there are different doped forms of hypoxanthine in the different guanine phases [26].

### 4.6. Fluorescence

**Figure 15:**
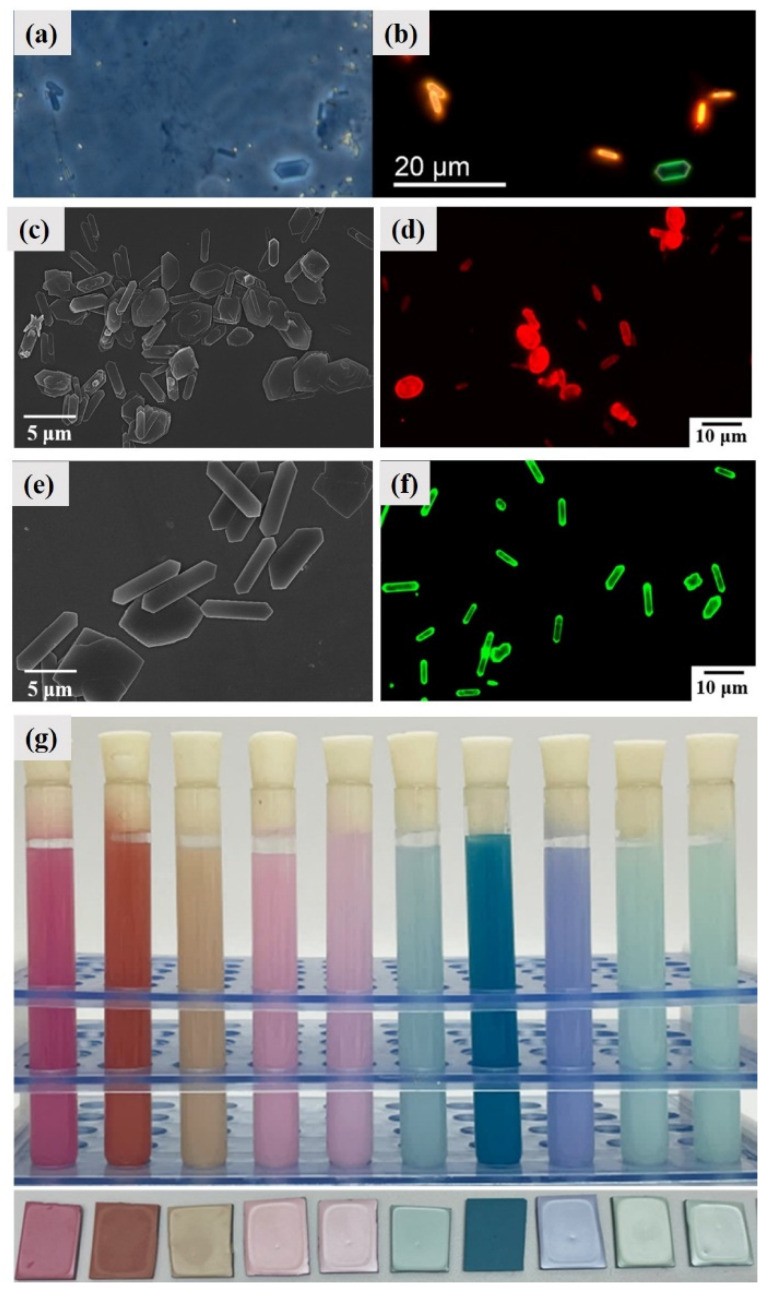
Fluorescence phenomena in biological guanine platelets and synthetic guanine platelets (AG β). (**a**) Phase contrast and (**b**) fluorescence light microscopy images of guanine platelets from the iris of T. delaisi. The regular guanine crystal at the bottom right has a weak blue-green fluorescence, as it lacks the red fluorescent pigment present in the other crystals. Shapes vary a lot in these crystals, but do not seem to differ systematically between fluorescent or non-fluorescent forms. Copyright 2014, BioMed Central [49]. SEM and fluorescence microscope images of synthesized (**c**,**d**) Nile red- and (**e**,**f**) fluorescein isothiocyanate-doped guanine platelets (AG β). Copyright 2023, Wiley-VCH [50]. (**g**) Colorful guanine pearlescent pigment dispersions and solids [51].

In aquatic environments where the ambient light is dominated by bluish light, red fluorescence can generate high-contrast signals. The cryptic fish *Tripterygion delaisi* has a bright red-fluorescent iris whose fluorescence can be rapidly up- and down-regulated in such environments [49]. The fluorescence signals are caused by guanine platelets from the iris which have a major red fluorescence and a weak blue-green fluorescence (Figure 15a,b). Due to the lack of fluorescence of guanine molecules and crystals, the formation of fluorescence in biological guanine platelets are caused by fluorescent molecule pigments doped into the guanine platelets. The controllable synthesis of β form guanine platelets with fluorescent properties and pearlescence was achieved by incorporating Nile red or fluorescein isothiocyanate (Figure 15c–f). The fluorescence intensities of the Nile red or fluorescein isothiocyanate were greatly enhanced after being doped into guanine crystals due to the inhibition of aggregation-caused quenching. A series of colorful guanine pearlescent pigment dispersions and solids were prepared based on different pigments that were doped into guanine platelets (Figure 15g).

### 4.7. Orientation and Assembly

**Figure 16:**
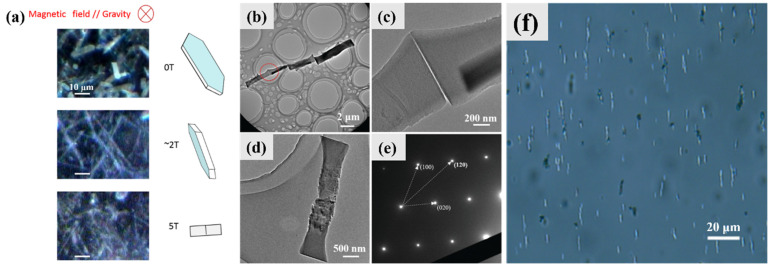
Orientation and assembly of guanine crystals. (**a**) Two-phase magnetic orientation of biogenic guanine platelets under magnetic fields up to 5 T oriented in the direction parallel to Earth’s gravity. Guanine platelets lying on a glass substrate with no magnetic field stand with their width oriented parallel to the direction of the ∼2 T magnetic field (//gravity), and stand with their length parallel to the direction of the 5 T magnetic field (//gravity). Copyright 2018, American Institute of Physics [52]. (**b**–**d**) TEM images of AG β microrod assembly, interface between two microrods, and individual microrod. (**e**) SAED pattern of the individual microrod shown in (**d**) in the neutralization precipitation system in formamide with pyrrole. (**f**) Light microscopy image of the oriented 1D assembly of AG β microrods under a magnetic field (1 T). Copyright 2021, Royal Society of Chemistry [53].

Biogenic guanine platelets exhibited a two-stage magnetic orientation when placed in a magnetic field that was increased up to 5 T (Figure 16a) [52]. Guanine molecules have a diamagnetic feature due to their anisotropic molecular structure. In the first stage, the widths of the horizontally deposited platelets aligned with the applied vertical magnetic field of ∼2 T (parallel to the direction of gravity). The second stage involved the alignment of the narrowest edge faces of the platelets in a direction parallel to the ∼5 T magnetic field. 

AG β microrods can be obtained in the neutralization precipitation system in formamide. The microrods can spontaneously form a one-dimensional (1D) assembly when dispersed in water. The obtained microrods with pyrrole as an additive can form a reversible 1D assembly by dispersing in different organic solvents and water. The two end faces of the microrods are smooth, which might be the reason why they can be attached to each other by the end-to-end mode and form the highly ordered 1D assembly (Figure 16b–d). The SAED pattern indicates that the long axis of the guanine microrod was parallel to the *a*-axis of AG β crystal, almost perpendicular to the (100) plane (Figure 16e). The main driving force for the 1D assembly is the hydrophobic attraction of the (100) planes of the guanine microrods. The oriented 1D assembly of guanine microrods with long axes perpendicular to the horizontal magnetic field can form in water under a 1 T magnetic field (Figure 16f).

## 5. Challenges in Synthetic Guanine Crystals

Highly reflective crystals of small organic molecules are not only composed of guanine, but also isoxanthopterin and 7,8-dihydroxanthopterin in a wide variety of optical systems in animals [3,54]. It is interesting to see how many organic biocrystals will be discovered and where they may be found.

For biological guanine, the research on the formation process and mechanism of guanine crystals in some organisms is in the early stages. There are many challenges in understanding guanine biomineralization. Why are guanine biominerals so different in different organisms? How are guanine molecules transported and which state is guanine in during transportation? How do organisms control the polymorphs and morphology of guanine? How do organisms organize and repair the multilevel structures of guanine biominerals? 

It is beautiful and fascinating to study artificial biominerals. The biomimetic synthesis of guanine platelets is impossible to obtain in neutral aqueous solutions directly or via an amorphous intermediate phase. This process is expected to produce commercial synthesized guanine platelets which can be used as green and safe pearlescent pigments. For macroscopic biomimetic synthesis, only guanine-based peptide nucleic acid monomers were used to prepare photonic crystals via self-assembly [55], and it is a challenge to build optical platforms based on guanine crystals in the laboratory.

## 6. Conclusions

The current knowledge on the fundamental physicochemical properties, polymorphs, morphology, and biomineralization principles of biological and synthetic guanine crystals were summarized in this work. Elaborate control of guanine crystals with multilevel structures is the key to obtaining excellent performances of biological guanine, which will be realized in the laboratory with more knowledge about guanine biomineralization. Guanine biomineralization principles will shed light on the design and synthesis of advanced organic materials including drugs, dyes, semiconductors, foods, etc., with specific polymorphs, sizes, morphologies, multilevel structures, and specified functions.

## Figures and Tables

**Table 1:** Aqueous solubility of guanine reported in the literature and obtained in our laboratory at 25 °C.

Solubility (μM)	Solvent	Polymorphs	Method	Ref.
39 ± 1	water	commercial guanine	LC ^1^	[12]
111 ± 4	citrate–phosphate buffer	commercial guanine	UV ^2^	[17]
25.4	water	commercial guanine	UV ^2^	[13]
42.6	water	commercial guanine	HPLC ^3^	[18]
13.9 ± 0.4	water	AG α	UV ^2^	unpublished data
16.5 ± 0.2	water	AG β	UV ^2^	
11.8 ± 0.2	phosphate-buffered solution	AG α	UV ^2^	
13.0 ± 0.1	phosphate-buffered solution	AG β	UV ^2^	

^1^ Liquid chromatography; ^2^ ultraviolet spectroscopy; ^3^ high-performance liquid chromatography.

**Table 2:** Crystal cell parameters of guanine polymorphs including guanine monohydrate (GM), dehydrated guanine monohydrate (dehydrated GM), anhydrous guanine α form (AG α), β form (AG β), and γ form (AG γ).

	Hydrated Guanine	Anhydrous Guanine			
	GM	Dehydrated GM	AG α	AG β	AG γ
Year	1971	2018	2006	2015	2015
CCDC number	GUANMH10	none	KEMDOW	KEMDOW01	none
Tautomer	keto-N9H	keto-N9H	keto-N7H	keto-N7H	keto-N7H
Temperature (K)	-	288			
Syngony	monoclinic	monoclinic	monoclinic	monoclinic	orthorhombic
Space	P2_1_/*n*	-	P12_1_/*c*1	P112_1_/*b*	P2_1_/m2_1_/n2_1_/*b*
*a*/Å	16.51	16.20	3.56	3.59	6.38
*b*/Å	11.28	10.99	9.65	9.72	9.73
*c*/Å	3.65	3.60	18.45	18.34	18.39
α	90	90	90	90	90
β	96.8	96.1	118.5	90	90
γ	90	90	90	119.5	90
Ref.	[27]	[15]	[5,28]	[5]	[5]

**Table 3:** Preparation methods of different guanine polymorphs.

Polymorph	Method	Ref.
GM	a. evaporation of a dimethylamine/water solution	[27]
	b. crystallization in an acidic aqueous solution	[15,20]
	c. crystallization in a neutral aqueous solution with surfactants	[15]
Dehydrated GM	dehydration of GM at 150 °C	[15]
AG α	a. saturated DMSO solution stored at 4 °C overnight	[29]
	b. neutralization precipitation in DMSO/water (4:1) with PVP ^1^ (guanine ~6 mM)	[30]
	c. neutralization precipitation in formamide with P(VP-co-VA) ^2^ and adenine or guanosine	[31]
	d. suspension in water at pH 2 and 90 °C	[6]
AG β	a. recrystallization of amorphous guanine	[16]
	b. neutralization precipitation in formamide with P(VP-co-VA) ^2^	[31,32]
	c. volatilization of guanine ammonia solution	[32,33]
	d. ammonia-induced crystallization of guanine-HCl solution at the air–water interface	[34]
	e. aqueous crystallization at pH 10	[6]
HAmG	rapid aqueous neutralization precipitation with high concentration guanine (~50 mM)	[16]

^1^ polyvinylpyrrolidone; ^2^ poly(1-vinylpyrrolidone-co-vinyl acetate).

## Data Availability

Not applicable.
